# Feasibility and Efficacy of a Parent-Focused, Text Message–Delivered Intervention to Reduce Sedentary Behavior in 2- to 4-Year-Old Children (Mini Movers): Pilot Randomized Controlled Trial

**DOI:** 10.2196/mhealth.8573

**Published:** 2018-02-09

**Authors:** Katherine L Downing, Jo Salmon, Trina Hinkley, Jill A Hnatiuk, Kylie D Hesketh

**Affiliations:** ^1^ Institute for Physical Activity and Nutrition School of Exercise and Nutrition Sciences Deakin University Burwood Australia

**Keywords:** child behavior, children, mHealth

## Abstract

**Background:**

Despite public health guidelines to limit sedentary behavior, many young children spend large amounts of time sedentary (eg, screen and sitting time) during waking hours.

**Objective:**

The objective of this study was to test the feasibility and efficacy of a parent-focused, predominantly text message–delivered intervention to support parents to reduce the amount of time their children spend in sedentary behavior.

**Methods:**

Mini Movers was a pilot randomized controlled trial delivered to parents of 2- to 4-year-old children in Melbourne, Australia. Participants were recruited through playgroups, social media, and snowball sampling. Eligibility criteria were having an ambulatory child (2-4 years), English literacy, and smartphone ownership. Participants were randomized to intervention or wait-list control on a 1:1 ratio after baseline data collection. The 6-week intervention was predominantly delivered via text messages, using a Web-based bulk text message platform managed by the interventionist. Intervention strategies focused on increasing parental knowledge, building self-efficacy, setting goals, and providing reinforcement, and were underpinned by the Coventry, Aberdeen & London-Refined taxonomy of behavior change techniques and social cognitive theory. The primary outcome was intervention feasibility, measured by recruitment, retention, intervention delivery, and fidelity; process evaluation questionnaires; and qualitative interviews with a subsample of participants. Secondary outcomes were children’s screen and restraint time (parent report), sitting time (parent report, *activ*PAL), and potential mediators (parent report). Linear regression models were used to determine intervention effects on secondary outcomes, controlling for the child’s sex and age and clustering by playgroup; effect sizes (Cohen's *d*) were calculated.

**Results:**

A total of 57 participants (30 intervention; 27 wait-list control) were recruited, and retention was high (93%). Process evaluation results showed that the intervention was highly acceptable to parents. The majority of intervention components were reported to be useful and relevant. Compared with children in the control group, children in the intervention group had significantly less screen time postintervention (adjusted difference [95% CI]=−35.0 [−64.1 to −5.9] min/day; Cohen's *d*=0.82). All other measures of sedentary behavior were in the expected direction, with small to moderate effect sizes.

**Conclusions:**

Mini Movers was shown to be a feasible, acceptable, and efficacious pilot intervention for parents of young children, warranting a larger-scale randomized control trial.

**Trial Registration:**

Australian New Zealand Clinical Trials registry: ACTRN12616000628448; https://www.anzctr.org.au/ Trial/Registration/TrialReview.aspx?ACTRN=12616000628448p (Archived by WebCite at http://www.webcitation.org/ 6wZcA3cYM)

## Introduction

Early childhood (ie, birth through 5 years) is recognized as a critical period in which sedentary behavior habits (eg, time spent sitting, screen time) are established [1,2]. In young children, sedentary behavior includes screen time, quiet play, and time spent in situations that restrict movement (eg, in car seats or prams). In early childhood, there is inconsistent evidence on the health and developmental outcomes associated with objectively assessed sedentary time (herein referred to as sedentary time) or time spent in situations that restrict movement (eg, in a car seat or pram). Some studies report no associations between sedentary time and adiposity [3,4] or psychosocial health [5], or between time spent restrained and motor development outcomes [6]. On the other hand, studies have reported unfavorable associations between girls’ total sedentary time and waist circumference [7] and between total percentage of time spent sedentary (for boys and girls) and locomotor skills [8]. For screen time, the evidence is more consistent. Television viewing, one of the most commonly studied sedentary behaviors in this age group, has been associated with unfavorable levels of adiposity and decreased psychosocial health and cognitive development [9,10], and total screen time has been associated with poorer well-being [11].

On the basis of these adverse health and cognitive outcomes, and given that some sedentary behaviors track over time [2], recommendations to limit sedentary behavior have been developed in several countries. These recommendations suggest that children aged 2 to 5 years should have less than 1 hour per day of screen time [12,13] and that situations that restrict movement, for example, in a car seat or pram, should be minimized for children aged 5 years and younger [12-14]. However, contrary to these recommendations, many young children are spending large amounts of time in these behaviors [6,15-18]. Feasible, acceptable, and effective interventions to reduce sedentary behaviors are therefore necessary during this early childhood period.

A systematic review and meta-analysis of interventions to reduce sedentary behavior during early childhood found that previous interventions can reduce both children’s screen time and sedentary time [19]. A majority of interventions included in that review were conducted in the preschool or child care setting, with comparatively few conducted in the home or in a community-based setting. However, subgroup analyses revealed that interventions conducted in the home setting, and including parent involvement, had the largest effects on screen time outcomes [19], suggesting this may be the most effective approach for modifying children’s screen behaviors. That review also highlighted the paucity of interventions targeting time spent in front of screens other than television or time spent restrained [19]. Furthermore, a limitation of existing interventions is that many, particularly those delivered to parents, have limited scalability (ie, the ability to be widely distributed at a population level). There is therefore a need to trial interventions that include parent involvement and have the potential for scalability and broad reach.

Population strategies that incorporate access to the home environment are challenging. In recognition of its potential reach, mobile phone technology is increasingly being used to deliver health behavior programs [20]. Text messages, or short message services, are particularly useful in this instance. They are a wide-reaching, low-cost channel for the delivery of health behavior programs and can be individually tailored, which has been shown to have positive effects on behavior change and to reduce attrition [21]. Few programs targeting child and adolescent health behaviors have used text messages to deliver intervention messages to parents [22], with only one targeting the early childhood population. Militello et al [23] conducted a pilot intervention using twice-weekly text messaging that focused on healthy lifestyle behaviors for parents of overweight and obese preschoolers. Results from that study showed significant improvements in parental knowledge regarding nutrition and physical activity. Additionally, the intervention was found to be feasible and acceptable for parents of young children [23], suggesting that this delivery mode holds promise in this population group. However, that intervention did not report on changes in children’s behaviors. No studies have utilized text messages to change sedentary behavior in this population; thus, it remains to be explored whether interventions delivered via text messages are feasible and can change sedentary behavior in this population. This study aimed to pilot test (1) the feasibility and (2) the potential efficacy behavior change strategies delivered predominantly by text message to support parents to reduce the amount of time their children spend in prolonged sedentary behavior.

## Methods

### Overview

This study was a two-arm pilot randomized controlled trial to evaluate a parent-focused, predominantly text message–delivered intervention to reduce sedentary behavior in 2- to 4-year-old children. The primary outcome was feasibility of the intervention. Secondary outcomes were changes in child sedentary behaviors (objectively assessed sitting time, and parent proxy-reported screen time) and potential mediators. The study protocol has been previously published [24] and is briefly outlined below. The study complied with the Consolidated Standards of Research Trials (CONSORT)-EHEALTH guidelines [25], including relevant items from the extension for pilot trials [26]. The Deakin University Human Research Ethics Committee granted ethics approval for the study (2016-103). This study was prospectively registered on May 16, 2016. Participants provided written, informed consent to participate on behalf of themselves and their children.

### Participants and Recruitment

Participants were recruited in Melbourne, Australia, through playgroups, social media (namely Facebook), and snowball sampling. In Australia, playgroups are informal gatherings for parents/caregivers and their children aged from birth to 5 years before the commencement of primary school. Snowball sampling included participating parents (recruited either through playgroups or on Facebook) passing on study information to friends and family (either hard copy flyers or by sharing information on Facebook). Inclusion criteria for parents were having an ambulatory child aged 2 through 4 years (ie, up to the age of 4.99 years); able to freely give informed consent; able to speak, read, and write fluent English; and smartphone ownership. The intervention was delivered to participants individually, regardless of recruitment method.

### Sample Size and Randomization

As the main outcome of this study was feasibility, no sample size power calculations were undertaken. Initially, this study aimed to recruit 100 participants. Participants were randomized to the intervention or wait-list control on a 1:1 ratio after baseline data collection. If more than one parent was recruited from a particular playgroup, randomization occurred at the group level to avoid potential contamination. A computer-generated random number schedule was developed by a researcher (not part of the research team) who had no contact with the participants. Group allocation was concealed in sealed, opaque envelopes, which were opened and revealed to the researcher and the participant(s) after baseline data collection to minimize selection and measurement bias. Participants were informed that they were either in Group 1 (intervention group; receiving the program immediately) or Group 2 (wait-list control group; receiving the program in 7 weeks).

### Mini Movers Intervention

The Mini Movers intervention was a predominantly text message–delivered intervention that aimed to provide parents with information and practical support to minimize the amount of time their children spend being sedentary and in screen time. The intervention was developed based on evidence-based guidelines for sedentary behavior in early childhood [12] and guided by the Coventry, Aberdeen & London-Refined (CALO-RE) taxonomy of behavior change techniques [27] and social cognitive theory [28]. Intervention strategies mapped to theoretical constructs are presented in the previously published study protocol [24]. Strategies focused on increasing parental knowledge, building self-efficacy, setting goals, and providing reinforcement. Participants in the intervention group received their intervention materials, including a Mini Movers information booklet, goal-checking magnet, and a Move and Play Every Day: National Physical Activity Recommendations for Children 0-5 Years brochure [12] either in person or by mail after baseline measures and allocation had been completed. The interventionist then had a one-on-one discussion with each participant individually, either in person or over the phone, to set their goals for the program. In total, 2 goals were set around reducing their child’s sedentary behavior; specifically, 1 screen time goal (eg, to limit their child’s screen time to 60 min or less per day) and 1 overall sedentary behavior goal (eg, to change an activity their child normally does sitting down, such as painting, to a standing activity). The goal-checking magnet aided participants to track their progress with their 2 goals for the duration of the program (6 weeks).

After the materials were given to participants and the goal-setting discussion was complete, the personalized, interactive text messages (ie, the main mode of intervention delivery) began the following day. Text messages were delivered using a Web-based bulk text message platform, managed by the interventionist. Participants received a welcome text message at the commencement of the program, followed by 3 standard text messages per week for 6 weeks (19 texts in total). The standard text messages included 2 behavioral messages with practical ideas and suggestions for limiting and displacing their child’s screen and sitting time, active play ideas, and monitoring and encouraging achievement of individual goals. Some text messages included links to reputable websites for further information.

The text messages were tailored to the participant’s name, child’s name, behavior goals, and the interventionist’s name. Participants were not required to respond to the text messages, with the exception of those texts used for goal monitoring, sent at the end of each week. These 2-way goal-monitoring text messages required participants to respond to let the interventionist know whether they had met their goal. On the basis of whether the response indicated the goals were achieved or not, parents were sent a predefined response, encouraging them to revisit their materials and keep trying the following week (if goals were not met) or congratulating them and encouraging them to keep going (if goals were met). Multimedia Appendix 1 shows examples of the types of text messages that were sent to participants.

### Wait-List Control

Participants randomized to the wait-list control group received the full intervention after postintervention assessments were completed.

### Measures

Data collection occurred pre- and postintervention. Measures included children’s height and weight (preintervention only), *activ*PAL (PAL Technologies Ltd, Glasgow, UK) accelerometers (worn for 7 days to objectively assess sitting time), and parent surveys.

### Primary Outcome

Intervention feasibility was measured by recruitment numbers, retention of participants, program metrics, and self-reported participant data, as described below.

#### Recruitment and Retention

Recruitment was measured by the proportion of contacted playgroups interested in the study (ie, the proportion of playgroups allowing a visit by the research team or distribution of flyers), the number of eligible parents within playgroups consenting, the number of parents recruited via social media and snowball sampling, and the time taken to recruit the sample. Retention was measured by the proportion of recruited participants providing measures at the end of the study.

#### Intervention Delivery and Fidelity

Intervention delivery and fidelity, that is, successful delivery to protocol, was measured by system reports (eg, delivered text messages) and auditing of protocol compliance in delivery of one-on-one goal-setting discussions by a single researcher.

#### Engagement in the Intervention and Acceptability

Engagement in the intervention was measured by the number of replies received from participants to the 2-way goal-monitoring messages and participant self-reported usage of and engagement with different components of the intervention, as reported in the postintervention survey. A subsample of randomly selected participants in the intervention group were invited to participate in qualitative telephone interviews (with a researcher other than the interventionist) to provide more detailed feedback about what they found useful and what they liked or disliked about components of the program. These participants were contacted after the program via mail and asked to return a separate consent form. Telephone interviews were scheduled for days and times convenient to the parents. Interviews included questions such as: “What did you find useful or most relevant to you about Mini Movers? How/why was that useful for you?”; “What did you think about the frequency of the text messages you received?”; and “How would you suggest we could improve the resources/materials so parents might be more likely to use them?”

### Secondary Outcomes

#### Children’s Objectively Assessed Sitting Time

Participating children wore an *activ*PAL for 7 consecutive days pre- and postintervention to objectively measure sitting time. The *activ*PAL has been shown to be valid, reliable, and feasible in young children [29]. The *activ*PAL was worn in the middle of the anterior aspect of the right thigh; monitors were sewn into purpose-made pouches affixed to leggings/bike shorts with Velcro, worn underneath normal clothes. Data were collected in 15-second epochs, and nonwear time was defined as 10 min of consecutive zero counts and removed from daily wear time. Children were asked to wear the monitors during waking hours (except for water-based activities such as bathing or swimming). To be included in analyses, children were required to have at least 6 hours of wear time on at least 4 days, including 1 weekend day. Nonwear time and minimum inclusion criteria were based on reliability criteria for ActiGraph (Pensacola, FL, USA) accelerometers [30], as no studies have examined reliability criteria for *activ*PAL accelerometers in this population. These criteria have been used previously in a pilot randomized control trial to reduce electronic media use in 2- to 3-year-old children [31].

#### Parent Proxy-Reported Sedentary Behavior and Screen Time

During each of the weeks that the children wore the *activ*PAL (ie, pre- and postintervention), parents completed Web-based surveys delivered via Qualtrics (Qualtrics Labs, Provo, UT). Parents with incomplete surveys (ie, missing responses) were followed up with an email and text message to prompt them to complete their survey. Parents reported their child’s usual time in the last week in a range of sedentary behaviors including sitting down for reading/quiet play/craft activities; situations that restrict movement (eg, in a car seat or stroller); and screen behaviors (ie, television viewing, computer and electronic games use, handheld electronic games use, smartphone use, and tablet computer use). Responses were open-ended (ie, hours and/or minutes per day). Parents also reported the number of days that their child watched television/DVDs or played video or computer games or used other electronic devices for entertainment for less than 1 hour (ie, met screen time recommendations). A 2-week test-retest reliability was conducted in a separate sample of 50 participants to test the reliability of these items (intraclass correlations=.07-.82 for continuous variables; kappa=.25 and percent agreement=52.3 for meeting recommendations question). Screen behaviors were examined individually as outcomes and also summed to give average daily minutes in total screen time (intraclass correlation=.98).

#### Potential Mediators

Parents were asked to report: their child’s preferences for sedentary behavior (sum of 3 items; 5-point Likert scale from *Never* to *Always*); their concerns about their child’s screen time use (sum of 4 items; 4-point Likert scale from *Strongly disagree* to *Strongly agree*); their use of screens to distract or occupy their child (sum of 6 items; 4-point Likert scale from *Never/rarely* to *All the time*); their views about screen time occupying children (sum of 4 items; 4-point Likert scale from *Strongly disagree* to *Strongly agree*); their self-efficacy for limiting sedentary behavior (sum of 5 items; 5-point Likert scale from *Not at all confident* to *Extremely confident*); logistic support for their child’s screen time (sum of 4 items; 5-point Likert scale from *Never or rarely* to *Several times each day*); and their beliefs/knowledge of screen time for young children (sum of 12 items; 4-point Likert scale from *Strongly disagree* to *Strongly agree*). The majority of these individual items had previously established reliability [32,33]. The reliability of new items was tested as described above; kappa=.22-.89 and percent agreement=33.4-97.7.

Internal reliability of all summed scores was tested using Cronbach alpha. Scores with reliability ≥.70 were included [34]. Of the 10 scales, 8 had acceptable reliability. The 2 remaining scales (child preferences for sedentary behavior=.64, and parental concerns about their child’s screen time use=.67) had moderate reliability; however, a decision was made to still include them as they made sense conceptually. Parents also reported their own frequency and duration in moderate- to vigorous-intensity physical activity (MVPA) in the previous week using the Active Australia Survey [35] and their usual week and weekend day television viewing [36], both collapsed to average minutes per day. Mediation analyses were not undertaken because of the small sample size.

#### Sample Characteristics and Child and Parent Adiposity

Parents reported their own and their child’s demographic information (eg, date of birth, parent education, parent employment status) and their child’s usual sleep duration (including daytime naps). Parents self-reported their height and weight, whereas children’s height and weight were measured before intervention by trained researchers using a Wedderburn portable rigid stadiometer, Wedderburn Tanita portable digital scales, and standardized measurement procedures [37,38]. Body mass index (BMI) was calculated by standard formula (weight in kilograms divided by height in meters squared); BMI categories (healthy weight, overweight, obese) were determined using age- and sex-specific international cutoff points for children [39] and World Health Organization’s classifications for parents [40].

### Statistical Analysis

All analyses were conducted using Stata 14 (StataCorp, College Station, TX, USA). Descriptive statistics were used to describe the baseline characteristics of the sample. Feasibility and acceptability were assessed using percentages and by analyzing qualitative data, as appropriate. Qualitative interviews were recorded, transcribed verbatim, and analyzed using NVivo (QSR International, 2002) qualitative software package. Participants’ responses to questions were coded to identify key themes. Linear mixed models were used to determine the effect of the intervention on the secondary outcomes (including children’s sedentary behavior and potential mediators), controlling for the child’s sex and age and clustering by playgroup. Given the small sample size, effect sizes (Cohen's *d*) were calculated. Values around .20 represent small, .50 moderate, and ≥.80 large effect sizes [41].

## Results

### Primary Outcome

#### Recruitment and Retention

**Figure 1:**
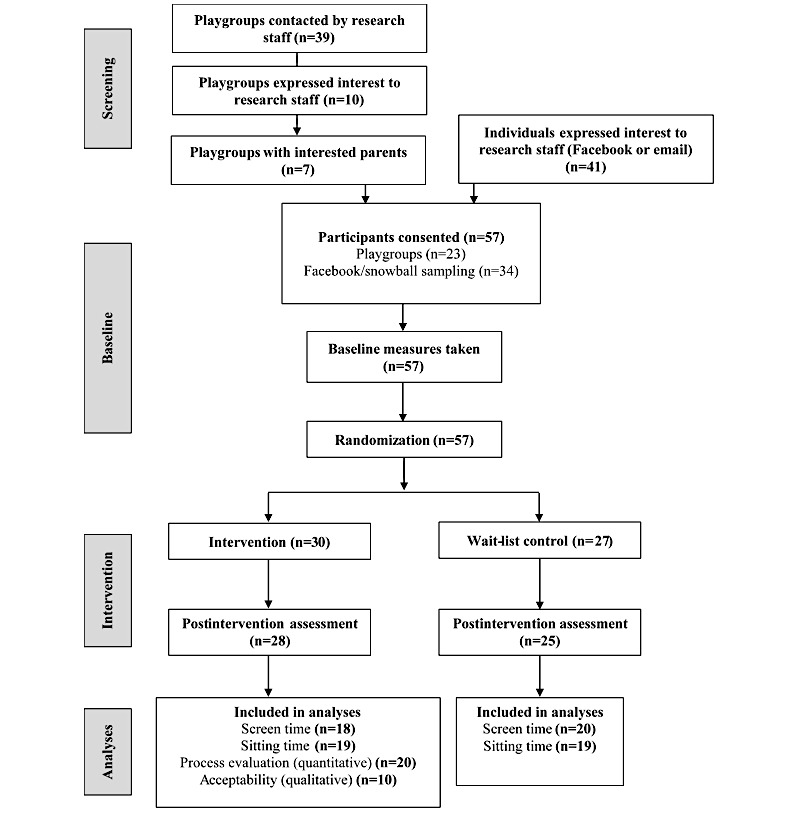
Trial flow diagram.

Recruitment was undertaken from June to October 2016. Figure 1 presents the flow of participants through the study. A total of 39 playgroup leaders were contacted initially. Of these, 10 leaders (26%) agreed to have a researcher visit the playgroup to talk to parents or put up flyers, 5 leaders (13%) declined participation, and 24 leaders (61%) did not respond (after a maximum of 2 emails and 2 phone calls). Of the 10 playgroups that received a recruitment visit, 7 had consenting parents (mean number of consenting parents per group=3.6, range=2-7; n=23 parents in total). A further 34 parents were recruited via Facebook and snowball sampling, resulting in a final sample of 57 participants who provided written, informed consent to participate in the study. Due to study time constraints, recruitment was planned for a set period of time (5 months) and was closed as planned, despite the recruitment target of 100 participants not being met.

All of the 57 consenting participants provided baseline data and were randomized to the intervention (n=30) or wait-list control (n=27) groups. One participant in the intervention group was uncontactable post baseline measures and hence did not receive the intervention; 1 participant from the intervention and 2 from the wait-list control group were uncontactable post intervention and hence did not provide follow-up data (93% retention). Acceptability questions were completed by 20 intervention participants postintervention. In total, 18 intervention (60%) and 20 (74%) control participants had complete proxy-reported child screen time data at both time points, and 19 participants from each group (63% and 70%, respectively) had valid *activ*PAL data at both time points and were included in efficacy analyses.

Child and parent characteristics are presented in Table 1. The average age of children was 3 years and just under half the sample were boys. One parent was the father of the child in the study and the remainder were mothers. The majority of parents were born in Australia, had a university degree, and were married/in a de facto relationship.

#### Intervention Delivery and Fidelity

The goal-setting discussions were all delivered; just over half (59%) were conducted in person with the remainder conducted over the phone. All of the standard text messages (ie, the welcome text message plus 2 behavioral and 1 goal-monitoring text message per participant per week; 19 text messages in total per participant) were also successfully delivered (n=551 text messages in total).

#### Engagement in the Intervention and Acceptability

Of the 174 goal-monitoring text messages sent in total, 145 (83.3%) received a response. Results of the self-reported usage of and engagement with the text messages, as well as perceived usefulness and relevance of different components of the intervention, are presented in Multimedia Appendix 2. The majority of participants (19/20; 95%) reported reading at least 9 of the 12 behavioral text messages. In terms of the 2 behavioral text messages that contained links to videos, 5 of the 20 participants (25%) reported watching none in full, 9 (45%) reported watching one of them in full, and 6 (30%) reported watching both in full. Five participants (25%) reported watching at least one of the videos more than once. In terms of the 5 behavioral text messages containing links to images or other websites, 1 participant (5%) clicked through to none, 11 participants (55%) clicked through to at least 3, and 5 (25%) clicked through to all 5 links. The majority of participants reported that the overall information, the goal planning, the booklet, and the text messages were very or extremely useful (10-13/20; 50%-65%) and very or extremely relevant (10-12/20; 50%-60%). Slightly fewer participants reported that the links to videos or other websites were very or extremely useful or relevant (both 9/20; 47%).

Of the 25 intervention participants invited to participate in the qualitative interviews, 10 participants provided written, informed consent (40% response). Interviews lasted 17 min on average. Overall, parents were very positive about the program:

I thought it was fantastic. We (the playgroup) were all really keen to participate, for the children...for their awareness and for our learning and I don’t have a criticism—I just thought it was lovely to promote... (an) active lifestyle and I think it’s really good that those things start young for children.

I thought it was a really great program. I think it had a lot of potential to really educate parents just about being aware of their kids’ activity and the consequences of inactivity...And it was very simple, like it wasn’t incredibly...complex or anything.

When asked about what components of the program they enjoyed specifically, many parents commented that the goal-setting was their favorite part. Parents thought that the goal-setting was particularly useful to keep them on track:

I think the thing that was most useful and I enjoyed the most was the goal-setting. So we had some goals around more physical activity in our day and also switching off the TV [television]...and so I liked being able to check off the goals and make sure that we met them every day.

Parents were also positive about the text messages, reporting that they were an easy and convenient way to receive the information. All parents reported that the frequency of receiving the text messages was acceptable; one parent suggested that they would have been happy to receive more (ie, 1 text message per day). Parents also liked the practical ideas and suggestions received in the text messages:

The information you gave around very practical ideas...rather than just sort of saying you know, they shouldn’t be sedentary and they shouldn’t be sitting and watching TV and screen time and things like that. You actually then provided alternatives...which I think sometimes as a parent, it’s not that you run out of ideas, but you do get stuck in old ways.

**Table 1:** Participant baseline characteristics.

Characteristics				Intervention (n=30)		Control (n=27)
**Child characteristics**						
	Sex (male), n (%)		15 (50)		11 (41)	
	Age in years, mean (SD)		3.2 (0.8)		2.9 (0.7)	
	Sleep duration in hours/day, mean (SD)		11.8 (1.1)		11.9 (1.0)	
	**BMI category, n (%)**					
		Healthy weight	24 (80)		20 (74)	
		Overweight	6 (20)		6 (22)	
		Obese	0 (0)		1 (4)	
	Siblings (yes), n (%)		20 (77)		16 (67)	
**Parent characteristics**						
	**Relation to child, n (%)**					
		Mother	26 (100)		23 (96)	
		Father	0 (0)		1 (4)	
	Age in years, mean (SD)		36.1 (3.9)		34.1 (3.7)	
	**BMI category, n (%)**					
		Healthy weight	14 (56)		18 (78)	
		Overweight	6 (24)		3 (13)	
		Obese	4 (20)		2 (9)	
	Born in Australia, n (%)		20 (77)		18 (78)	
	**Education level, n (%)**					
		Year 12 or equivalent	1 (4)		0 (0)	
		Trade/certificate/diploma	1 (4)		6 (26)	
		University degree/postgraduate	24 (92)		17 (74)	
	**Marital status, n (%)**					
		Never married	0 (0)		1 (4)	
		Married/de facto	26 (100)		22 (96)	
	**Work status, n (%)**					
		Maternity/paternity leave	9 (35)		7 (30)	
		Student	1 (4)		0 (0)	
		Home duties full time	4 (15)		7 (30)	
		Part-time work	12 (46)		6 (26)	
		Full-time work	0 (0)		3 (13)	

^a^BMI: body mass index.

When prompted about the links in the text messages, some parents reported that they only clicked through a few of them. All parents were positive about the content of the links, but some reported that they often did not have time to click through and then would forget to go back:

A couple of times I couldn’t (click through) at the time, on my phone, for whatever reason...but they were all quite good actually...the ones that I saw. There was a couple I certainly didn’t delve into ’cos I either forgot to go back to it...or at the time I couldn’t access it so I’d sort of put it on the backburner and then...the next week evolved I suppose.

When asked whether they thought the program had changed the way they do things in their family, parents commented that the program had made them more conscious of screen and sedentary time, and in some cases had other flow-on effects such as spending more time with their children:

I do tend to spend more time with the kids...because one of the goals was to reduce TV time, I have found that I do spend more time with them. So I will try and keep the TV reduced as much as possible, like switched off as long as I possibly can. And yeah, I do end up spending more time playing with them because you know, I want him to stand and I want him to move around and things like that.

It definitely made me rethink TV time...and use it a bit more sparingly I guess, instead of a babysitter.

We’ve definitely increased physical activity levels in our kids and we’re walking to kinder, and we’re walking to the shops a lot more and we’re relying on the car a lot less...And...we kind of had iPads, but we’ve pretty much decommissioned our iPads now so they’re not existing in our house anymore and we just switch off the TV a lot more. So that’s definitely been a sustained effect of the program.

There were also some suggestions from parents on how to improve the program. Some parents suggested that a website or Facebook page would be beneficial as a central place for all of the information provided. One parent also suggested that Facebook would be useful for allowing parents in the program to chat to each other. Some parents also thought that revisiting their goals halfway through the program may have been beneficial:

Maybe...for the first few weeks start off with a more lenient goal and then make your way to a more...a stricter goal to yourself.

Finally, some parents reported that although they liked the premise of the program, they found that the information provided was not necessarily new to them and that they already did many of the things suggested:

The text messages, maybe for people who weren’t active, would be a good reminder to be active...(but) the suggestions weren’t particularly relevant for me...like we already did a lot of that stuff.

I walk the dogs 7 days, every morning...she walks with me or she’s in the trike, we can be gone for half an hour or an hour each morning. And then she’ll come with me to the gym and then we’ll do...another gym training class where mums and the kids are there in a big hall, and the kids just jump around the whole time. And then we do swimming another day...so I guess that I feel like over the week, there’s activity every day...um, there’s play with other children, there’s awareness...there’s a focus on us being out. So, I didn’t feel our lives were very sedentary before the program.

**Table 2:** Baseline and postintervention values, adjusted differences, and effect sizes for sedentary behavior outcomes.

Outcome variable (all min/day unless otherwise specified)			Baseline, mean (95% CI)				Post intervention, mean (95% CI)				Adjusted mean difference (95% CI)^a^			Effect size (Cohen's *d*)		
			Control		Intervention		Control		Intervention						
**Parent reported**															
	Total screen time^b^		92.0 (68.1 to 115.9)	109.7 (78.2 to 141.3)		99.5 (69.2 to 129.8)		79.2 (53.2 to 105.1)			−35.0 (−64.1 to −5.9)			0.82	
	TV/DVD viewing		77.5 (57.5 to 97.5)	88.1 (54.9 to 121.2)		78.0 (57.4 to 98.6)		69.2 (43.1 to 95.2)			−15.0 (−34.3 to 4.3)			0.61	
	Computer/e-game^c^ use		0.0 (0.0 to 0.0)	0.6 (−0.6 to 1.7)		0.0 (0.0 to 0.0)		0.0 (0.0 to 0.0)			—			—	
	Handheld e-game use		0.0 (0.0 to 0.0)	0.0 (0.0 to 0.0)		0.0 (0.0 to 0.0)		0.0 (0.0 to 0.0)			—			—	
	Smartphone use		4.8 (0.1 to 9.4)	5.9 (1.3 to 10.4)		5.8 (−1.0 to 12.5)		3.5 (−0.5 to 7.6)			−1.9 (−7.2 to 3.4)			0.38	
	Tablet use		10.3 (0.02 to 20.5)	15.0 (2.8 to 27.2)		7.1 (−2.0 to 16.2)		6.7 (1.5 to 11.9)			−8.2 (−23.0 to 6.6)			0.21	
	Time restrained		63.2 (39.6 to 86.9)	74.7 (46.2 to 103.2)		64.3 (49.7 to 78.8)		57.5 (37.3 to 77.7)			−16.2 (−39.3 to 7.0)			0.48	
	Time sitting		127.3 (82.5 to 172.0)	126.7 (97.8 to 155.5)		118.5 (83.3 to 153.7)		106.1 (75.2 to 137.0)			−13.5 (−63.4 to 36.4)			0.15	
	Days/weekdays child has <1 hour screen time		3.5 (2.4-4.6)	3.6 (2.3 to 4.9)		3.6 (2.6 to 4.6)		3.4 (2.2 to 4.7)			−0.1 (−1.7 to 1.4)			0.11	
**activPAL**															
	Sitting time		265.8 (212.4-319.2)	281.7 (223.6 to 339.9)		262.1 (209.6 to 314.6)		256.0 (205.6 to 306.3)			−22.3 (−80.8 to 36.3)			0.26	

^a^Adjusted for child sex, child age, and clustering by playgroup.

^b^Sum of individual screen behaviors.

^c^e-game: electronic game.

### Secondary Outcomes

#### Children’s Sedentary Behavior

Table 2 presents the mean minutes per day parents reported their children spent in each of the individual screen behaviors, total screen time, and time spent restrained and sitting, as well as *activ*PAL assessed sitting time, at baseline and post intervention. 

Adjusted mean differences between intervention and control groups were all in the expected direction (favoring the intervention group), with a significant difference seen for child total screen time only. Intervention participants reduced their total screen time by 30.6 min/day (from 109.7 to 79.2 min/day), whereas screen time for control participants increased by 7.5 min/day (from 92.0 to 99.5 min/day; *d*=0.82). Reductions in individual screen behaviors resulted in small to medium effect sizes (*d*=0.21-0.61). Time spent restrained was reduced in the intervention group by 17.2 min/day (from 74.7 to 57.5 min/day) and increased in the control group by 1.0 min/day (from 63.2 to 64.3 min/day; *d*=0.48). Parent-reported sitting time was reduced in both the intervention and control groups, by 20.6 min/day (from 126.7 to 106.1 min/day) and 8.8 min/day (from 127.3 to 118.5 min/day), respectively (*d*=0.15). Sitting time, as measured by *activ*PAL, was reduced in the intervention group by 25.8 min/day (from 281.7 to 256.0 min/day) and in the control group by 3.7 min/day (from 265.8 to 262.1 min/day; *d*=0.26).

#### Potential Mediators

Changes in potential mediators from baseline to post intervention for the intervention and control groups are reported in Table 3. The largest effect (*d*=0.93) was seen for parental logistic support for their child’s screen time (eg, putting the television on for their child, buying DVDs), with a significant adjusted mean difference between intervention and control groups post intervention. Moderate effects were also seen for parent MVPA (not in the expected direction; *d*=0.66), parental views about the use of screen time for occupying children (*d*=0.61), and parental self-efficacy to limit their child’s sedentary behavior (*d*=0.43).

**Table 3:** Baseline and postintervention values, adjusted differences, and effect sizes for potential mediators.

Outcome variable	Baseline mean (95% CI)		Postintervention mean (95% CI)		Adjusted mean difference (95% CI)^a^	Effect size (Cohen's *d*)
	Control	Intervention	Control	Intervention		
Child preferences for sedentary behavior (eg, more likely to watch TV than be active); possible range, 0 to 12	3.5 (2.4 to 4.5)	3.8 (3.0 to 4.6)	3.4 (2.6 to 4.1)	3.2 (2.4 to 4.1)	−0.5 (−1.6 to 0.6)	0.26
Parental concerns about child’s screen time (eg, child watches too much TV); possible range, −8 to 8^b^	−4.8 (−6.1 to −3.5)	−4.0 (−5.2 to −2.8)	−5.4 (−6.5 to −4.3)	−5.4 (−6.3 to −4.6)	−0.9 (−2.4 to 0.5)	0.40
Parent use of screens to distract or occupy child (eg, uses TV to distract child when he/she is being difficult); possible range, 0 to 18	3.5 (2.2 to 4.8)	4.4 (2.6 to 6.1)	3.0 (1.7 to 4.3)	3.4 (1.6 to 5.3)	−0.8 (−2.1 to 0.4)	0.23
Parental views about screen time occupying children (eg, has difficulty getting child to eat without screens as distraction); possible range, −8 to 8^c^	−4.5 (−6.1 to −2.8)	−3.2 (−5.3 to −1.0)	−4.7 (−6.2 to −3.1)	−4.8 (−6.7 to −2.9)	−1.3 (−2.8 to 0.2)	0.61
Parental self-efficacy to limit child’s sedentary behavior; possible range, 0 to 20	14.8 (13.6 to 15.9)	12.9 (11.0 to 14.9)	14.8 (13.5 to 16.0)	14.2 (12.6 to 15.7)	1.2(−0.5 to 2.9)	0.43
Parental logistic support of screen time (eg, number of times in the last week parent put the TV on for child); possible range, 0 to 20^c^	5.3 (3.8 to 6.7)	5.8 (4.1 to 7.6)	5.3 (3.5 to 7.2)	3.9 (2.3 to 5.5)	−1.7 (−3.0 to −0.4)	0.93
Parental beliefs/knowledge of child screen time (eg, TV is educational for children); possible range, −24 to 24^d^	2.6 (−3.0 to 8.2)	2.3 (−2.3 to 6.8)	1.7 (−3.1 to 6.5)	3.1 (−2.2 to 8.4)	3.0 (−0.7 to 6.8)	0.27
Parent moderate- to vigorous-intensity physical activity (min/day)	27.1 (12.0 to 42.2)	38.2 (−20.3 to 96.6)	43.2 (25.4 to 61.1)	41.2 (−4.6 to 87.0)	−16.6 (−35.7 to 2.6)	0.66
Parent TV viewing (min/day)	70.3 (38.4 to 102.1)	91.8 (52.1 to 131.5)	64.1 (44.9 to 83.3)	83.2 (57.5 to 108.9)	6.8 (−21.5 to 35.2)	0.05

^a^Adjusted for child sex, child age, and clustering by playgroup.

^b^Lower score indicates fewer concerns.

^c^Lower score indicates more favorable outcome.

^d^Lower score indicates parental beliefs/knowledge consistent with evidence.

## Discussion

### Principal Findings

This study aimed to test the feasibility and efficacy of a parent-focused, predominantly text message—delivered intervention to support parents to minimize the amount of time their children spend in sedentary behavior. Results show that the intervention was largely feasible and acceptable to parents of young children. The study also showed a statistically significant and meaningful reduction in children’s total screen time in the intervention group compared with the control group, with promising results for the other secondary outcomes.

Recruitment was particularly difficult through playgroups compared with the other recruitment strategies utilized in this study (eg, social media). Initial contact with playgroup leaders was challenging; many did not reply to multiple phone calls or emails. Leaders who declined participation (n=5) cited reasons, including participation in other research, their playgroup potentially disbanding, or simply that they were not interested. Within playgroups, there was also evidence of peer influence, whereby if 1 or 2 parents were very interested initially, it would often prompt other parents to read the information and potentially consent to participating. Conversely, if no one initially expressed interest, then other parents would not consent. Future studies may benefit from exploring other recruitment avenues in this population. In particular, Facebook seemed to be a useful platform for recruiting parents in this study. This is consistent with reports of recruitment from other studies. For instance, an mHealth intervention delivered to parents of infants (<3 months) targeting infant feeding practices recruited more than 50% of the intervention group online (compared with around 30% recruited by practitioners and 7% recruited face-to-face by researchers) [42]. This suggests that Web-based methods may be more appealing to parents of young children, perhaps given that they are able to read about the study and consent in their own time. Despite these difficulties, and although recruitment targets were not met, a sufficient sample was recruited for a pilot study. Previous feasibility studies targeting screen time in this population have included similar or smaller samples [31,43]. Moreover, despite the small sample, a significant reduction in total screen time was observed and effect sizes showed favorable effects.

The acceptability of the intervention overall was high. In both the quantitative process evaluation and the qualitative phone interviews, parents reported that the goal-setting and the text messages were very useful and relevant. Many parents noted that the goal-planning magnet was useful to help keep them on track. It has been suggested that higher parental compliance with behavior change techniques such as goal-setting and self-monitoring results in better child outcomes [44]. It was encouraging that a number of parents reported in the qualitative interviews that they had continued to try to meet their goals and that the changes in their families were sustained once the intervention ended. However, parents reported using the text messages containing links to images and other websites less frequently and also reported finding them less useful and relevant, compared with the goal-setting and behavioral text messages. Parents of young children are likely to be time-poor, and, as some parents noted in qualitative interviews, if they were not able to click through immediately, they would often forget to go back. A pilot text message intervention focusing on healthy lifestyle behaviors for parents of overweight and obese preschoolers reported that parents wanted a short, easy-to-read, and strong message [23]. It may be that providing links to more information or to videos may not be necessary or feasible in this population.

The efficacy results are also encouraging. In addition to the statistically significant reduction and large effect in total screen time in the intervention group compared with the control group, a moderate effect was seen for television viewing. Given that television viewing constitutes around 80% of total screen time in this sample and in previous studies [15], it is important that interventions target this behavior. An intervention conducted in preschools reported very similar results, with a significant reduction in total screen time of almost 30 min a day but no effect on television viewing [45]. A home-based intervention reported a significant reduction in television viewing in the intervention compared with control group of 37 min a day; however, that intervention specifically targeted television viewing rather than total screen time [46]. Small effects were seen for smartphone use and tablet use in this study; however, use of these screens was relatively low compared with television viewing, leaving little scope to reduce those behaviors. It may be that specific strategies are needed to target children’s use of these newer devices. Although the effect size was small, it was promising to see a reduction in objectively assessed sitting time of more than 20 min per day in the intervention group compared with the control group. A previous intervention targeting only screen time use found no effect on objectively assessed sitting time [31] and suggested that specific strategies should be included to target reductions in sitting time. Results from this study support this, showing that, by providing parents with strategies to reduce sitting time, potentially positive outcomes can be observed.

There was a significant reduction in parental logistic support for screen time (eg, putting the television on for the child) in the intervention group compared with the control group. This suggests that the strategies used in the intervention were effective at changing parents’ behavior around their child’s screen time. Potentially, the practical strategies around alternatives to screen time may have resulted in this change; in qualitative interviews, some parents reported that they switched off the television more and used it less as a babysitter. Moderate effects were also seen for parental views about screen time occupying children and parental self-efficacy to limit their child’s sedentary behavior. This is particularly promising given that the intervention was theoretically based on the social cognitive theory [28], in which there is a strong focus on self-efficacy. Previous cross-sectional studies have reported that higher parental self-efficacy is associated with lower amounts of screen time in preschool-aged children [47-49], suggesting that future interventions would benefit from continuing to target self-efficacy as a mediator of children’s screen time.

There was also a moderate effect on parent’s self-reported MVPA; however, the adjusted mean difference was in the unexpected direction, in that parents in the intervention group reduced their MVPA by almost 17 min per day compared with the control group. A possible explanation for this is that many parents set their overall sedentary behavior goal as walking to local destinations without the pram (ie, to decrease their child’s time spent restrained). As a result, in trying to achieve this goal by having their child walk more, the parents themselves may have ended up walking more slowly than usual. Future research should consider objectively measuring parents’ physical activity to examine potential changes in sedentary time and light-intensity physical activity, in addition to MVPA.

### Strengths and Limitations

Limitations of this study include the small sample size and the number of participants without full outcome data. This is mostly because of parents not completing, or only partially completing, Web-based surveys, despite reminders to do so. It may be that Web-based surveys are not practical for parents of young children, as there is more opportunity for them to be distracted or forget to come back to it. Additionally, a number of children did not have valid *activ*PAL data. Although the *activ*PAL accelerometers (sewn into a pouch and affixed to leggings) were predominantly acceptable for the children and parents, many parents noted that they often forgot to put the leggings back on after naps or bathing. This may have resulted in fewer valid hours of wear time on particular days, potentially excluding them from analyses.

It was a reasonably homogenous sample with a high percentage being very highly educated (>75% with a university degree or higher). Although over-representation of higher-educated women in research is common [50,51], the outcomes observed in this study may not have been observed in a sample of parents with lower educational attainment. Finally, intervention fidelity may have been somewhat compromised as a number of parents reported, both quantitatively and qualitatively, that they did not click through to all of the links provided in the text messages. Many parents also reported that they did not watch the videos provided in these links in full, suggesting that different strategies may be needed for some parents to increase compliance. However, given that a significant intervention effect was seen for children’s screen time, the text messages alone may have been sufficient to elicit behavior change and the links may not have been necessary.

There are also a number of strengths of this study. Comprehensive measures of sedentary behavior were included, including parent proxy report of specific screen-based behaviors, time spent restrained, and sitting time, in addition to children’s objectively assessed sitting time. The intervention was developed based on the social cognitive theory [28] and targeted specific behavior change mediators from the CALO-RE taxonomy of behavior change techniques [27]. Interventions are more likely to be effective if they are theory-based [52] and are closely aligned with behavior change techniques [53].

### Conclusions

Mini Movers was found to be a feasible and acceptable intervention for parents of 2- to 4-year-old children. Moreover, child sedentary behavior was reduced, suggesting that the intervention was efficacious. It will be important for future studies to measure individual screen behaviors; results from this study support previous findings that although at this age screen time consists largely of television viewing, there is some evidence of use of smartphones and tablets and so targeting these behaviors specifically in interventions may be efficacious. The findings and learnings from this pilot study show sufficient promise to inform the development of a future large-scale trial adequately powered to determine impacts on children’s sedentary behavior and to explore the mediators of behavior change. If effective, the main delivery mode (ie, text messages) means that this intervention has the ability to be scaled up and widely disseminated.

## Appendix

Multimedia Appendix 1 Examples of text messages.

Multimedia Appendix 2 Parent self-reported usage of and engagement with text messages and perceived usefulness and relevance of the intervention.

Multimedia Appendix 3 CONSORT-EHEALTH checklist (V 1.6.1).

## Abbreviations

BMI: body mass index
MVPA: moderate- to vigorous-intensity physical activity
