# Supplementary material for: Feasibility and Efficacy of a Parent-Focused, Text Message–Delivered Intervention to Reduce Sedentary Behavior in 2- to 4-Year-Old Children (Mini Movers): Pilot Randomized Controlled Trial
Source: JMIR Mhealth Uhealth. 2018 Feb 9;6(2):e39. doi: 10.2196/mhealth.8573 (PMC5889816; doi:10.2196/mhealth.8573)
Supplement: Multimedia Appendix 1 [file mhealth_v6i2e39_app1.pdf]

Appendix 1. Examples of text messages

Type of text message	Example content[a]
Behavioural	Annie, get Josh to help make some playdough! Here’s a great recipe with no cooking required: <link to recipe>. Remember, encourage Josh to stand up while playing with it! Katherine – Mini Movers
Goal-checking	Hi Carolyn, how did you go sticking with your goals to limit Sienna’s screen time to 60 mins a day and to do puzzles standing up instead of sitting 2 days this week? Text me back YES if you achieved them or NO if you weren’t able to this week. Katherine – Mini Movers
Goal-checking – response to YES reply	Great to hear! Remember how good you feel achieving your goals – bottle that feeling & use it as motivation on tough days. Keep it up! Katherine – Mini Movers
Goal-checking – response to NO reply	It’s common to slip up sometimes Julia. The important thing is trying again next week! Use your Mini Movers Goal Checker magnet to keep you on track. Katherine – Mini Movers

[a] Names have been changed for anonymity
