# Supplementary material for: Feasibility and Efficacy of a Parent-Focused, Text Message–Delivered Intervention to Reduce Sedentary Behavior in 2- to 4-Year-Old Children (Mini Movers): Pilot Randomized Controlled Trial
Source: JMIR Mhealth Uhealth. 2018 Feb 9;6(2):e39. doi: 10.2196/mhealth.8573 (PMC5889816; doi:10.2196/mhealth.8573)
Supplement: Multimedia Appendix 2 [file mhealth_v6i2e39_app2.pdf]

Appendix 2. Parent self-reported usage of and engagement with text messages and perceived usefulness and relevance of the intervention

Table 1. Self-reported usage of and engagement with behavioural text messages (n=20)

Considering the behavioural text messages…	11-12 texts n (%)	9-10 texts n (%)	7-8 texts n (%)	5-6 texts n (%)	3-4 texts n (%)	1-2 texts n (%)	None n (%)
How many did you read	17 (85.0)	2 (10.0)	0 (0.0)	0 (0.0)	1 (5.0)	0 (0.0)	0 (0.0)
How many gave you ideas that you used with your child	0 (0.0)	2 (10.0)	5 (25.0)	4 (20.0)	4 (20.0)	4 (20.0)	1 (5.0)

Table 2. Self-reported usage of and engagement with text messages containing links to videos (n=20)

Considering the text messages with links to videos…	2 texts n (%)	1 text n (%)	None n (%)
How many did you watch in full	6 (30.0)	9 (45.0)	5 (25.0)
How many did you watch in part	2 (10.0)	5 (25.0)	13 (65.0)
How many did you watch more than once	2 (10.0)	3 (15.0)	15 (75.0)
How many gave you ideas that you used with your child (n=19)	4 (21.5)	5 (26.3)	10 (52.6)

Table 3. Self-reported usage of and engagement with text messages containing links to images and other websites (n=20)

Considering the text messages with links to images or other websites…	5 texts n (%)	4 texts n (%)	3 texts n (%)	2 texts n (%)	1 texts n (%)	None n (%)
How many did you click through to the links	5 (25.0)	4 (20.0)	2 (10.0)	7 (35.0)	1 (5.0)	1 (5.0)
How many did you click through to more than once	0 (0.0)	0 (0.0)	1 (5.0)	3 (15.0)	3 (15.0)	13 (65.0)
How many gave you ideas that you used with your child	1 (5.0)	0 (0.0)	7 (35.0)	5 (25.0)	4 (20.0)	3 (15.0)

Table 4. Perceived usefulness of components of the Mini Movers intervention (n=20)

How **useful** was/were…	Extremely<br>n (%)	Very<br>n (%)	Moderately<br>n (%)	Slightly<br>n (%)	Not at all<br>n (%)
The information overall	2 (10.0)	8 (40.0)	8 (40.0)	2 (10.0)	0 (0.0)
The goal planning	3 (15.0)	9 (45.0)	5 (25.0)	3 (15.0)	0 (0.0)
The booklet	1 (5.0)	9 (45.0)	6 (30.0)	4 (20.0)	0 (0.0)
The text messages	4 (20.0)	9 (45.0)	2 (10.0)	4 (20.0)	1 (5.0)
The links to videos (n=19)	3 (15.8)	6 (31.6)	4 (21.1)	4 (21.1)	2 (10.5)
The links to other websites (n=19)	2 (10.5)	7 (36.8)	6 (31.6)	2 (10.5)	2 (10.5)

Table 5. Perceived relevance of components of the Mini Movers intervention (n=20)

How **relevant** was/were…	Extremely<br>n (%)	Very<br>n (%)	Moderately<br>n (%)	Slightly<br>n (%)	Not at all<br>n (%)
The information overall	2 (10.0)	8 (40.0)	7 (35.0)	2 (10.0)	1 (5.0)
The goal planning	4 (20.0)	9 (45.0)	4 (20.0)	2 (10.0)	1 (5.0)
The booklet	0 (0.0)	11 (55.0)	2 (10.0)	7 (35.0)	0 (0.0)
The text messages	2 (10.0)	8 (40.0)	3 (15.0)	6 (30.0)	1 (5.0)
The links to videos (n=19)	1 (5.3)	8 (42.1)	3 (15.8)	5 (26.3)	2 (10.5)
The links to other websites (n=19)	1 (5.3)	8 (42.1)	5 (26.3)	3 (15.8)	2 (10.5)
